# Synthesis of Novel Isoindolinones: Carbonic Anhydrase Inhibition Profiles, Antioxidant Potential, Antimicrobial Effect, Cytotoxicity and Anticancer Activity

**DOI:** 10.1002/jbt.70261

**Published:** 2025-04-14

**Authors:** Yusuf Serdar Yazıcıoğlu, Şeydanur Elmas, Zeynep Kılıç, Murat Çelik, Buket Bakan, Ufuk Atmaca, Songül Bayrak

**Affiliations:** ^1^ Department of Molecular Biology and Genetics, Faculty of Sciences Atatürk University Erzurum Turkey; ^2^ Department of Chemistry, Faculty of Sciences Atatürk University Erzurum Turkey

**Keywords:** anticancer activity, antimicrobial and antioxidant activity, carbonic anhydrase, enzyme inhibition, isoindolinone, sulfamate

## Abstract

An efficient one‐pot method has been developed for synthesizing novel isoindolinone derivatives from 2‐benzoylbenzoic acid using chlorosulfonyl isocyanate and alcohols. This reaction occurs under mild, metal‐free conditions, rendering it a sustainable and effective approach for isoindolinone synthesis. The inhibitory potential of the synthesized compounds against human carbonic anhydrase (hCA) I and II isozymes was evaluated and compared with the standard inhibitor, acetazolamide (AAZ). Additionally, their antimicrobial and antioxidant activities were assessed using various bioanalytical methods, with results benchmarked against standard reference compounds. Furthermore, cytotoxicity and anticancer activity were investigated in L929 and A549 cell lines via the WST‐1 assay following a 24 h exposure. Among the synthesized derivatives, compounds **2c** and **2f** exhibited superior inhibitory effects on hCA I and II compared to AAZ, with Ki values ranging from 11.48 ± 4.18 to 16.09 ± 4.14 nM for hCA I and 9.32 ± 2.35 to 14.87 ± 3.25 nM for hCA II. These findings indicate that compounds **2c** and **2f** have a high affinity for the enzyme's active site, resulting in more effective inhibition of its catalytic activity. Compound 2e emerged as the most promising candidate, demonstrating potent carbonic anhydrase inhibition and significant antioxidant and antimicrobial properties. None of the synthesized compounds displayed cytotoxic effects on healthy cells at the tested concentrations. Additionally, compound **2a** exhibited dose‐dependent anticancer activity against A549 cells. These results suggest that isoindolinone derivatives, particularly **2f**, hold substantial potential for further pharmaceutical development as multifunctional bioactive agents.

## Graphical abstract

Highly effective one‐pot synthesis of novel isoindolinones Inhibitory, antioxidant, antimicrobial cytotoxicity, and anticancer potentials of synthesized novel isoindolinone derivatives were investigated.

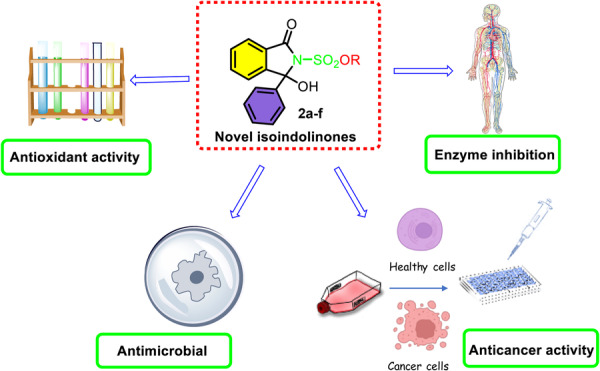

## 1 Introduction

**Figure 1:**
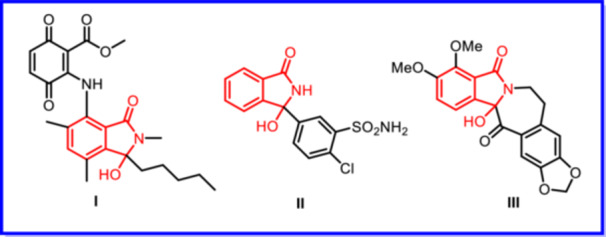
Bioactive isoindolinone nucleus.

The 3‐hydroxyisoindolinone framework represents a significant and versatile structure in synthesizing natural products and pharmaceutical compounds [1, 2]. These compounds exhibit substantial biological activity and are present in various bioactive molecules with drug‐like properties. Notable examples include isoindolinone‐containing compounds such as Corollosporine derivatives **(I),** which possess antibacterial properties [3], Chlortalidone **(II),** an antihypertensive drug [4], and Chilenine **(III),** a natural product with pharmacological relevance [5] (Figure 1).

Traditionally, synthesizing 3‐hydroxyisoindolinone derivatives has depended on metal‐catalyzed annulation or acylation of functionalized benzoic acid derivatives [6, 7, 8]. However, these metal‐catalyzed processes have significant limitations, including high costs, complexity, and the necessity for meticulous removal of metal residues, especially in pharmaceutical applications. Therefore, developing an efficient and practical synthetic methodology for 3‐hydroxyisoindolinone derivatives under mild and sustainable conditions remains essential.

CSI is widely recognized in organic chemistry as a versatile reagent with various applications [9]. It has been used in multiple transformations, including the synthesis of β‐lactam derivatives [10, 11], the preparation of sulfonamides, [2 + 2] cycloadditions with alkenes, the synthesis of the Burgess reagent, and the conversion of alcohols into carbamates [12]. This study presents an efficient one‐pot approach for synthesizing novel isoindolinone derivatives. This reaction proceeds under mild conditions, eliminates the need for metal catalysts, and achieves good yields in the presence of TFA and CSI.

Carbonic anhydrases (CAs) are zinc‐dependent enzymes crucial for various cellular processes, including pH balance, respiration, and metabolic pathways. These enzymes facilitate the reversible conversion of carbon dioxide (CO₂) into bicarbonate, playing a key role in physiological functions. In humans, 15 distinct carbonic anhydrase isoforms have been identified, each with specific functions and unique tissue distributions [13]. Human carbonic anhydrases (hCAs) participate in essential processes such as CO₂ regulation, gas exchange, electrolyte secretion, and biosynthetic reactions. Additionally, specific isoforms, like CA IX and CA XII, are associated with cancer progression [14]. CA IX is significantly increased by hypoxia and is highly expressed in solid tumors, including colon, glioma, and breast cancers. CA XII, first recognized as a tumor‐associated isoform in 1998, is overexpressed in malignant tissues and plays a significant role in tumor development [15, 16].

The CAI isoenzyme is associated with glaucoma, a serious eye condition marked by optic nerve degeneration and increased intraocular pressure [17]. It is a leading cause of blindness worldwide. When applied topically, human carbonic anhydrase inhibitors, such as dorzolamide and brinzolamide, effectively lower intraocular pressure [18]. However, their notable side effects point to the need for new treatment alternatives. Because carbonic anhydrases regulate intraocular pressure by controlling aqueous humor production, carbonic anhydrase inhibitors (CAIs) have been extensively researched for treating glaucoma [19].

In recent years, dual‐target drugs, also known as chimeric drugs, have garnered attention in medicinal chemistry. By simultaneously inhibiting multiple enzymes involved in disease progression, these drugs provide advantages such as overcoming drug resistance and minimizing adverse effects [20]. Moreover, reactive oxygen species (ROS), continuously generated in the body, contribute to cellular damage and play a crucial role in various age‐related diseases, including cancer, diabetes, glaucoma, cardiovascular diseases, and neurodegenerative disorders. The excessive accumulation of ROS, known as oxidative stress, worsens disease progression [21]. Antioxidants, by scavenging ROS, reduce oxidative damage and present therapeutic potential.

Currently, most cancer therapies consist of cytotoxic agents that indiscriminately target both cancerous and healthy cells, resulting in severe side effects [22]. An ideal anticancer drug should selectively focus on cancer cells while minimizing toxicity to healthy tissues. Enzyme inhibition, antimicrobial, and antioxidant research remain fundamental in drug discovery, as they help identify compounds with promising pharmacological properties. Isoindolinones, a class of molecules with diverse biological activities, are often used in drug design [23]. This study explores the carbonic anhydrase inhibitory, antioxidant, antimicrobial, and anticancer properties of novel isoindoline compounds, which show significant potential for future drug development efforts.

## 2 Results and Discussion

Initially, Ethyl 1‐hydroxy‐3‐oxo‐1‐phenylisoindoline‐2‐sulfonate (**2a**) was synthesized using chlorosulfonyl isocyanate in dichloromethane as the solvent, under acidic conditions with trifluoroacetic acid at room temperature. Compound **2a** was obtained with an excellent yield of 95%. Its structure was confirmed through characterization by ^1^H and ^13^C‐NMR, IR, and high‐resolution mass spectrometry (HRMS).

**Figure 2:**
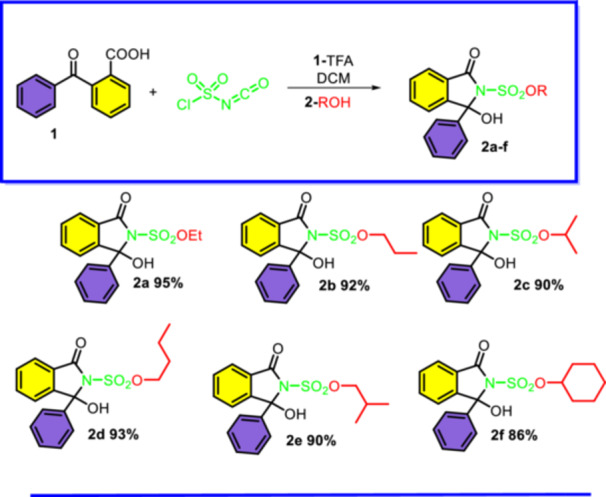
Substrate scope for synthesis of novel isoindolinone derivatives ^a,b^. ^a^Reaction conditions: 1.0 eq of 2‐benzoylbenzoic acid, 1.1 eq of CSI, catalytic amount of TFA, 10 mL DCM, reaction was stirred for 2 h and 1 mL ROH for 1 h, ^b^Isolated yield.

The substrate scope of this transformation was investigated and the results are summarized in Figure 2. Notably, the reaction of 2‐benzoylbenzoic acid with CSI and various alcohol derivatives proceeded smoothly to furnish *n*‐propanol derivative **2b** (92%), 2‐propanol derivative **2c** (90%), *n*‐butanol derivative **2d** (93%), 2‐methyl‐propan‐1‐ol derivative **2e** (90%), and cyclohexanol derivative **2f** (86%), respectively (Figure 2).

## 3 Enzyme Inhibition

**Figure 3:**
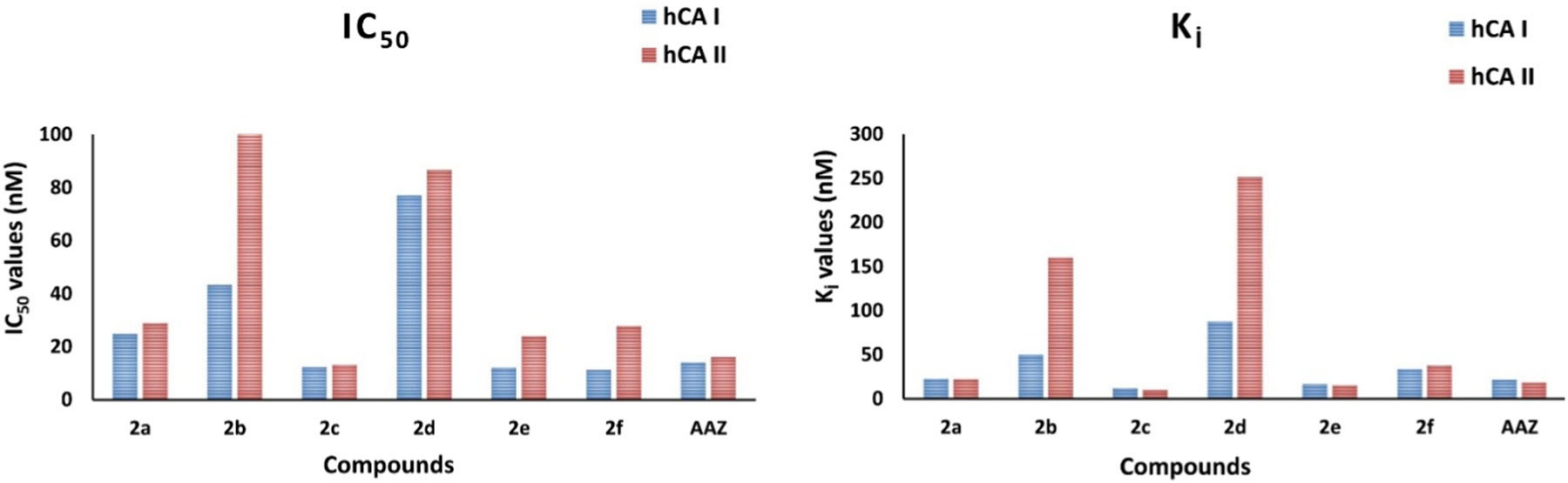
IC₅₀ values and Ki constants for hCA I and hCA II for isoindoline compounds (**2a‐f**) and standard AAZ.

Carbonic anhydrase (CA) enzymes, metalloenzymes vital to physiological pH regulation and various biological processes, were the focus of this study. Specifically, the inhibitory effects of novel synthesized isoindolinone derivatives on hCA I and II isoenzymes were investigated. Given the critical role of hCA II in intraocular pressure regulation, these compounds were considered potential candidates for glaucoma treatment. Initial inhibition studies assessed the effects of **2a‐f** against hCA I and hCA II isoenzymes, with results summarized in Table 1 and Figure 3.

**Table 1:** Inhibition effects of isoindoline compounds (2a–f) on hCA I and hCA II isoenzymes.

[a] AZA was used as a standard inhibitor for hCA‐I and hCA‐II.						
IC_50_ (nM)						K_İ_ (nM)
Compounds	hCA I	*r* ^2^	hCA II	*r* ^2^	hCA I	hCA II
**2a**	24.29 ± 1.980	0.9861	28.84 ± 0.151	0.9906	22.03 ± 9.21	21.69 ± 10,56
**2b**	42.01 ± 1.347	0.9830	231 ± 1	0.9841	49.49 ± 12.07	160.34 ± 46.59
**2c**	12.56 ± 0.370	0.9898	13.02 ± 0.041	0.9964	**11.48** ± **4.18**	**9.32** ± **2.35**
**2 d**	75.73 ± 1.205	0.9855	86.37 ± 0.338	0.9917	87.08 ± 35.21	251.48 ± 37.07
**2e**	11.84 ± 0.132	0.9977	23.85 ± 0.152	0.9835	33.32 ± 15.11	37.54 ± 14.66
**2 f**	11.24 ± 0.291	0.9906	27.80 ± 0.170	0.9818	**16.09** ± **4.14**	**14.87** ± **3.25**
**AAZ**	13.74 ± 0.652	0.9828	15.62 ± 0.375	0.9878	20.89 ± 1.728	18.16 ± 0.882

[a] AAZ was used as a standard inhibitor for hCA‐I and hCA‐II.

The novel synthesized isoindolinone derivatives (**2a‐f**) demonstrated significant potential as potent hCA inhibitors. These compounds exhibited high affinity for hCA I and hCA II isoforms, with low nanomolar inhibitory activities. The Ki values for hCA I ranged from 11.48 ± 4.18 to 87.08 ± 35.21 nM, while IC₅₀ values ranged from 11.24 ± 0.291 to 75.73 ± 1.205 nM. For hCA II, the Ki values ranged from 9.32 ± 2.35 to 160.34 ± 46.59 nM, and the IC₅₀ values ranged from 13.02 ± 0.041 to 231 ± 1 nM (Table 1).

All synthesized isoindolinone derivatives exhibited potent inhibitory activity against hCA I and II enzymes, as indicated by their Ki values, which reflect a high affinity for the enzymes.

In the rational design of these novel inhibitors, the molecular framework was structured based on a modular approach involving a head, core, linker, and tail strategy. The core structure of the synthesized molecules consists of an isoindolinone scaffold, which serves as the fundamental pharmacophore contributing to enzyme interaction. The head portion incorporates a sulfonyl functional group known for its strong coordination with carbonic anhydrases’ active site zinc ion. The linker region, varied across the synthesized compounds, includes alkyl and cyclic alcohol derivatives (e.g., 2‐propanol, cyclohexanol, *n*‐butanol), influencing the binding affinity and selectivity towards hCA I and II isoforms. The tail functionalization involves different alkyl and aryl moieties that modulate the lipophilicity and steric effects, thereby impacting the overall enzyme binding dynamics.

Upon analysis of the results, it was determined that the 2‐propanol (**2c**) and cyclohexanol (**2f**) derivatives, synthesized through the reaction of 2‐benzoyl benzoic acid with CSI and various alcohol derivatives, were the most effective inhibitors of the hCA I enzyme, with Ki values of 11.48 ± 4.18 nM and 16.09 ± 4.14 nM, respectively, outperforming the standard inhibitor AAZ. The ethyl group‐containing derivative 2a (Ki: 22.03 ± 9.21 nM) displayed inhibitory activity comparable to AAZ. In contrast, **2b** (Ki: 49.49 ± 12.07 nM) and **2f** (Ki: 33.32 ± 15.11 nM) showed weaker inhibitory effects against hCA I than AAZ. The *n*‐butanol **(2d)** derivative (Ki: 87.08 ± 35.21 nM) exhibited the lowest inhibitory activity among the tested compounds.

For the physiologically dominant hCA II, compounds **2c** (Ki: 9.32 ± 2.35 nM) and **2e** (Ki: 14.87 ± 3.25 nM) showed the strongest inhibitory effects, similar to those observed for hCA I. In contrast, compound **2d** (Ki: 251.48 ± 37.07 nM) showed the weakest inhibitory effect. The attachment of *n*‐propanol (**2b**) instead of *n*‐butanol (**2d**) to the nitrogen on the isoindoline ring increased the activity by 1.56‐fold. Furthermore, the Ki value for AAZ used as a positive standard carbonic anhydrase inhibitor, was measured as 18.16 ± 0.882 nM. 2a (Ki: 21.69 ± 10,56 nM) showed an activity comparable to AAZ, similar to its behavior in hCA I.

In the literature, Atmaca and colleagues investigated the effects of synthesized isoindoline derivatives on various enzymes, including butyrylcholinesterase, acetylcholinesterase, α‐glucosidase, and carbonic anhydrase (CA) I and II, which are linked to diseases such as type 2 diabetes, Alzheimer's disease, glaucoma, and epilepsy. Their findings demonstrated that these compounds exhibited potent inhibitory activity against these enzymes at the micromolar (μM) level [23]. In a separate study, Nadaroğlu and colleagues synthesized isoindoline‐1,3‐dione derivatives and examined their inhibitory effects on different isoforms of human carbonic anhydrases (hCA, EC 4.2.1.1). This study assessed the inhibition of cytosolic CA I and II, membrane‐bound CA IV, and tumor‐associated CA IX. The results indicated that oxime and sulfonamide derivatives showed significant selective activity against the targeted hCA IX compared to the more widely expressed hCA I and II [24]. Another study focused on the synthesis, characterization, and biological evaluation of isoindoline‐1,3‐dione‐based oxime and benzenesulfonamide hydrazone compounds. Hydroxyiminoethyl aromatic derivatives (10–18 series) were evaluated as potential zinc‐binding agents for an understudied functional group in carbonic anhydrase (CA, EC 4.2.1.1) inhibition. The synthesized compounds were tested for their inhibitory effects on hCA I, hCA II, hCA IV, and hCA IX isoforms. The findings revealed that oxime and sulfonamide derivatives exhibited selective inhibitory properties against hCA IX, with distinct inhibitory ranges and ratios differentiating the two subsets [25]. Gundogdu and colleagues synthesized isoindole‐1,3‐dione‐substituted sulfonamide derivatives and evaluated their hCA isoenzyme inhibitory activity. Their study demonstrated that the synthesized compounds exhibited potent inhibitory activity, with Ki constants ranging from 7.96 to 48.34 nM, compared to acetazolamide (AAZ) (Ki = 436.20 nM for hCA I; Ki = 93.53 nM for hCA II) [26]. These findings highlight the significant pharmaceutical and industrial potential of isoindolinone derivatives.

## 4 Antioxidant Activity Results of Isoindolinone Compounds

**Figure 4:**
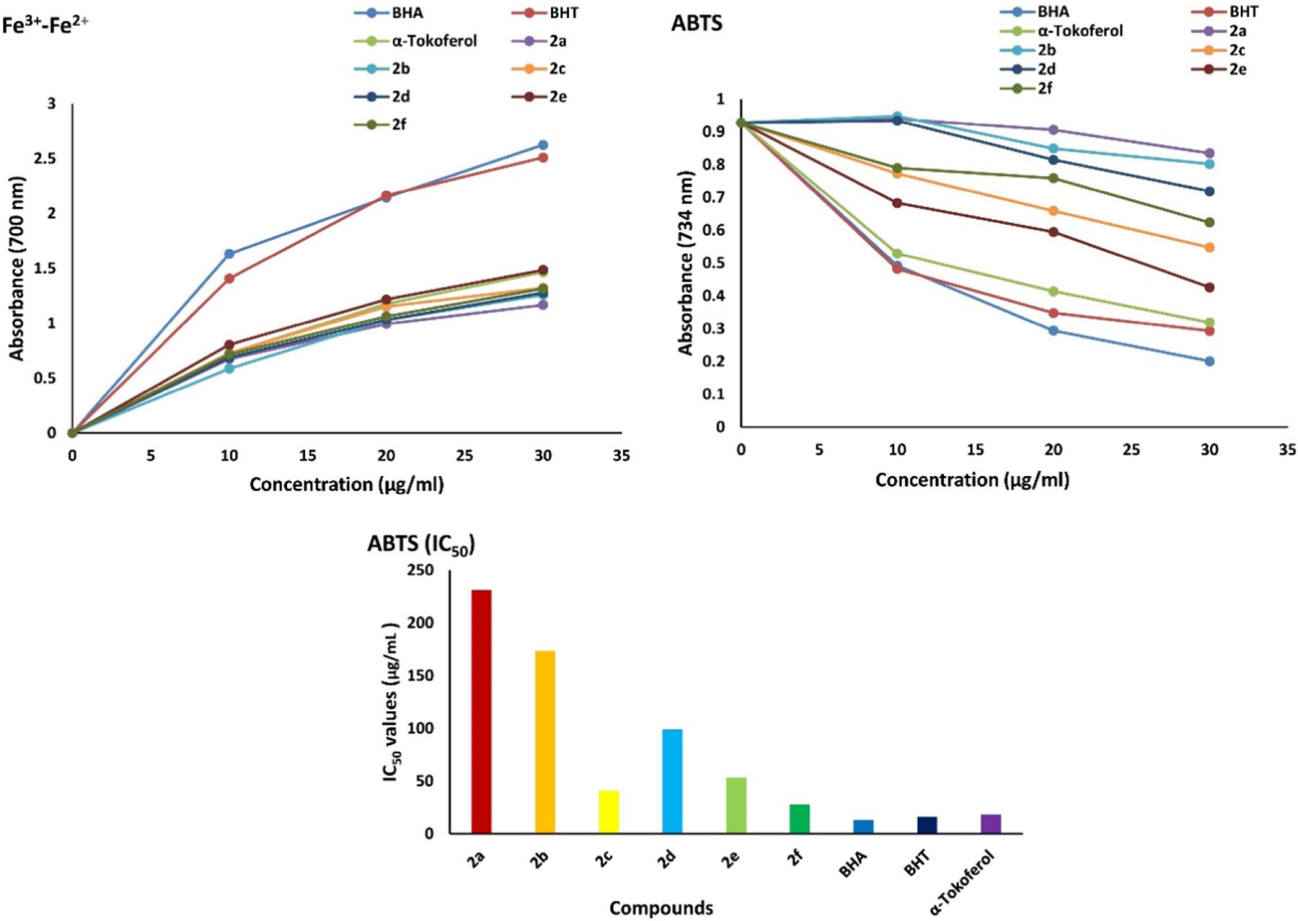
ABTS+ radical scavenging activities of isoindolinone compounds (**2a‐f**) and standard antioxidants by the Fe^+2^‐Fe^+3^ method and determination of half maximum concentration (IC_50_) for reducing the power of 30 μg/mL concentration.

Antioxidants are crucial components of the body's defense systems, protecting cells from damage caused by free radicals, particularly reactive oxygen species (ROS). While ROS are naturally produced during cellular metabolism, excessive amounts can lead to oxidative stress. This condition can damage biomolecules, including cell membranes, proteins, and DNA, contributing to cellular dysfunction and diseases like diabetes, cancer, immunodeficiency, and obesity. Antioxidants offer an effective defense by neutralizing ROS and mitigating oxidative stress, even at low concentrations [27]. The antioxidant capacities of isoindolinone derivatives were evaluated using two distinct methods: ABTS cation radical scavenging activity and Fe^+2^‐Fe^+3^ reduction capacity. As presented in Figure 4 and Table 2, all tested compounds demonstrated moderate to low antioxidant activity compared to standard antioxidants, including BHT, BHA, and α‐tocopherol, which were used as references.]

**Table 2:** Determination of reducing the power of isoindoline compounds (**2a‐f**) and standard antioxidants by Fe^+2^‐Fe^+3^ reduction and ABTS•+ radical scavenging methods at 30 μg/mL concentration.

Compounds	Fe^3+^‐Fe^2+^ reducing (λ700)	*R2*	ABTS radical scavenging (λ734)	*R2*
**2a**	1.168 ± 0.058	0.918	0.835 ± 0.024	0.641
**2b**	1.260 ± 0.079	0.965	0.802 ± 0.072	0.757
**2c**	1.323 ± 0.133	0.965	0.549 ± 0.049	0.999
**2d**	1.278 ± 0.059	0.925	0.719 ± 0.032	0.829
**2e**	1.316 ± 0.023	0.944	0.624 ± 0.040	0.947
**2f**	**1.485** ± **0.156**	0.940	0.426 ± 0.132	0.977
**BHA**	2.625 ± 0.241	0.941	0.201 ± 0.040	0.983
**BHT**	2.510 ± 0.111	0.923	0.293 ± 0.094	0.897
**α‐Tokoferol**	1.465 ± 0.284	0.960	0.319 ± 0.101	0.935

Results from ABTS cation radical scavenging and Fe^+2^‐Fe^+3^ reduction experiments conducted at 30 µg/mL concentrations showed consistent trends for isoindoline compounds. When absorbance values were compared with standard antioxidants, the order was as follows: BHA > BHT > α‐Tocopherol > **2f** > **2c** > **2e** > **2 d** > **2b** > **2a**. The IC_50_ values calculated for the ABTS method were as follows: BHA (12.83 µg/mL) > BHT (16.11 µg/mL) > α‐Tocopherol (18.23 µg/mL) > **2f** (27.72 µg/mL) > **2c** (40.76 µg/mL) > **2e** (53.30 µg/mL) > **2d** (99.00 µg/mL) > **2b** (173.25 µg/mL) > **2a** (231.00 µg/mL).

The study investigated the impact of substituents attached to the nitrogen atom in the isoindolinone ring on the antioxidant activity of the compounds. The findings revealed compound **2f**, which contains a cyclohexanol group, exhibited the highest antioxidant activity, whereas compound **2a**, containing an ethyl group, showed the lowest activity. This suggests that the cyclohexanol group in compound **2f** plays a critical role in the radical scavenging mechanism, likely due to its electron‐donating properties, which facilitate interaction with and neutralization of radical species. Additionally, the length and branching degree of the alkyl chains in compounds containing *n*‐propanol (**2b**), 2‐propanol (**2c**), *n*‐butanol (**2d**), and 2‐methyl‐propan‐1‐ol (**2e**) were observed to influence antioxidant activity. In general, compounds with branched alkyl chains demonstrated higher antioxidant activity.

While the antioxidant activity of isoindolinone derivatives is scarcely documented in the literature, several studies have investigated structurally related 2‐indolinone compounds. For instance, Afşah et al. investigated the ABTS radical scavenging activities of 2‐indolinone bis(Mannich base) derivatives [28]. They noted that these compounds exhibited antioxidant activity at varying levels, ranging from low to high.

## 5 Antibacterial and Antifungal Activity Results of Isoindolinone Compounds

**Figure 5:**
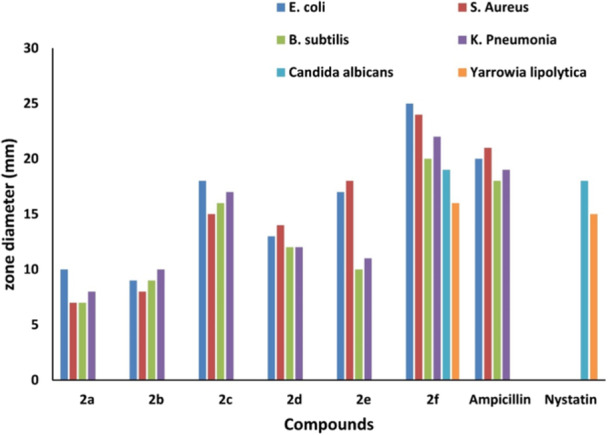
Determination of antimicrobial properties of isoindoline compounds (**2a‐2f**) at a concentration of 1000 μg/mL by antibacterial and antifungal methods.

The antifungal and antibacterial activities of the isoindolinone derivatives (**2a–f**) were evaluated against six different bacterial and fungal strains, as shown in Figure 5 and Table 3. To assess the antimicrobial properties of the synthesized compounds, inhibition zones (measured in mm) were determined for *Staphylococcus aureus, Bacillus cereus, Klebsiella pneumoniae, Escherichia coli, Candida albicans*, and *Yarrowia lipolytica* and subsequently analyzed.

**Table 3:** Disk diffusion results of isoindolinone derivatives (2a‐f).

Bacteria/Fungal		Compounds^a^						Negative control^b^		Positive control^c^
		2a	2b	2c	2d	2e	2f		DMSO	S
**GR(+) bacteria**	**B1**	7	11	16	12	10	20		—	18
	**B2**	7	8	19	14	13	24		—	21
**GR(‐) bacterica**	**B3**	9	10	18	13	14	25		—	20
	**B4**	8	10	17	12	11	22		—	19
**Fungal**	**F1**	—	—	—	—	—	19		—	18
	**F2**	—	—	—	—	—	16		—	15

Abbreviations: B1, Bacillus subtilis; B2, *Staphylococcus aureus*; B3, *Escherichia coli*; B4, Klebsiella pneumonia; F1, *Candida albicans*; F2, Yarrowia lipolytica; GR+, GR positive; GR−, GR negative; ‐, inhibition zone not formed.

The inhibitory zone (mm) diameter for different concentrations (µg mL^−1^).

Disc diameter: 5 mm

^a^ Compounds: Each at a concentration of 1000 μg/mL.

^b^ Negative Control: DMSO (10%).

^c^ Positive Control: Antibacterial Standard Drug, S; Ampicillin (10 µg disc^−1^), Antifungal Standard Drug, S; Nystatin (10 µg disc^−1^).

The evaluation of the antimicrobial activities of the synthesized isoindolinone derivatives revealed that compound **2f** exhibited a broader spectrum and more potent antimicrobial activity against both Gram‐negative and Gram‐positive bacteria, as well as fungi, compared to the other compounds. The enhanced activity of this compound is attributed to the presence of the cyclohexanol group in its structure. The cyclohexanol group is hypothesized to increase cell membrane permeability by forming hydrogen bonds with the imidazole, thiol, carboxyl, and amino groups of proteins in bacterial and fungal cell membranes. This interaction likely results in the leakage of intracellular components, ultimately leading to cell death.

In contrast, substituting the cyclohexanol group with an ethyl group in compound **2a** significantly reduced its antimicrobial activity. This decrease is explained by the weaker interaction of the ethyl group with cell membranes due to its hydrophobic nature, thereby diminishing its antimicrobial effect. **2b**, **2c**, **2d**, and **2e**, containing *n*‐propanol, 2‐propanol, *n*‐butanol, and 2‐methyl‐propan‐1‐ol groups displayed comparable antimicrobial activities. Compound **2c** demonstrated activity comparable to the standard antibiotic ampicillin against certain bacteria. This suggests that while the length and branching of alkyl chains have a limited impact on antimicrobial activity, other structural features of the compounds also play a significant role in determining their efficacy.

### 5.1 In Vitro Cytotoxicity and Anticancer Activity

**Figure 6:**
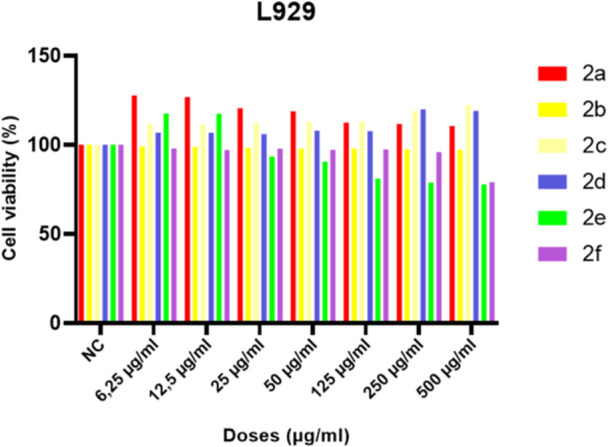
Cytotoxicity test results of test compounds after 24 h exposure at different concentrations in L929 cell lines by using WST‐1 assay. Data are presented as mean ± SD from three independent replicate experiments.

Evaluation of the safety profile of new candidate pharmacological formulations is essential before their incorporation into biomedical applications. In that case, cytotoxic effects were examined to determine the dose‐response of compounds in L929 cells by using WST‐1 test. According to test results, differences in cell viability were observed between the compounds, but no statistically significant cytotoxic effect was observed at applied concentrations. Even, the **2a**, **2c** and, **2d** compounds were increased cell proliferation when compared with control group (Figure 6).

**Figure 7:**
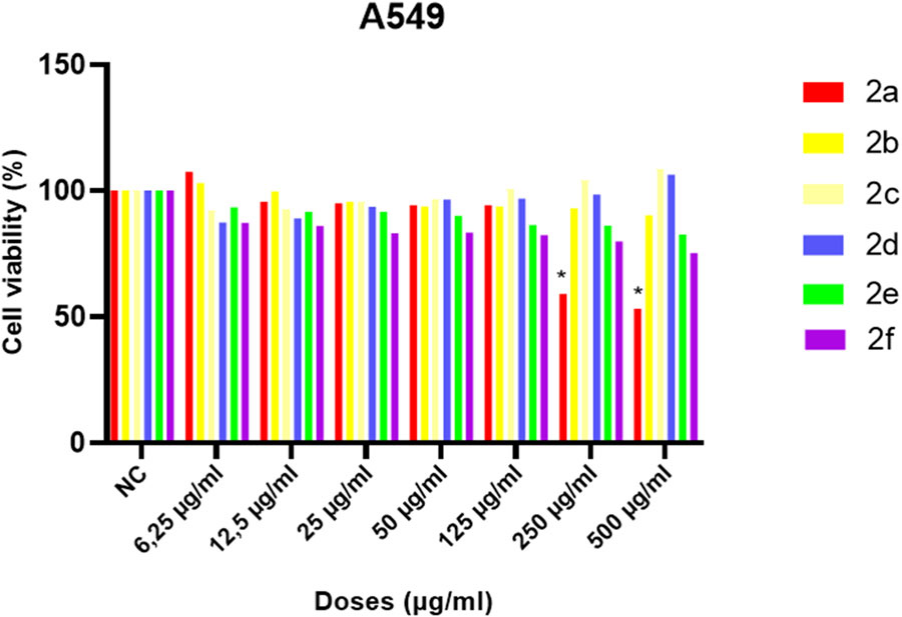
Anticancer activity test results of test compounds after 24 h exposure at different concentrations in A549 cell lines. Data are presented as mean ± SD from three independent replicate experiments. *p < 0.0001.

As part of research to develop new active anticancer agents that facilitate the attainment of a novel pharmacological profile, the assay was performed on human lung cells which is one of the most common causes of death in the world. The assays were utilized to calculate the dose–response parameters of anticancer activity for compounds against the A549 cell line across a wide range. The outcomes for each compound are presented as the percentage of treated cells relative to untreated control cells. The anticancer efficacy was evaluated by revealing that one compound (**2a**) demonstrated inhibitory effects at high doses against A549 lung tumor cells at dose‐dependent as presented in Figure 7. The 50% growth inhibitory concentration (IC_50_) value for **2a** compound was calculated as 650,250 µg/ml.

Similar anticancer studies have been conducted in the literature on isoindolinone and other synthesized compounds. These studies evaluate the biological activity of various derivatives, highlighting the potential anticancer effects of isoindolinone and related structures. Specifically, they detail the inhibitory effects on cell proliferation, regulatory roles in apoptotic mechanisms, and cytotoxic activities against specific cancer cell lines [29, 30].

## 6 Conclusions

In conclusion, we synthesized novel isoindolinone derivatives from 2‐benzoylbenzoic acid using CSI and various alcohols (ROH) in good yields. This method holds significant promise for the practical synthesis of isoindolinone derivatives with potential applications in pharmaceutical and industrial settings. The approach is particularly advantageous due to its mild reaction conditions, short reaction times, and environmentally friendly, metal‐free design.

Among the synthesized isoindolinone compounds, **2c** and **2f** exhibited superior inhibition of hCA I and hCA II isozymes compared to acetazolamide, suggesting their potential as effective carbonic anhydrase inhibitors. Compound **2f** demonstrated a multifunctional pharmacological profile, combining potent carbonic anhydrase inhibition with significant antioxidant and antimicrobial properties. This observation highlights a possible mechanistic interplay between these biological activities, where carbonic anhydrase inhibition may contribute to broader therapeutic effects, potentially enhancing oxidative stress regulation and antimicrobial defense. Given the widespread therapeutic applications of carbonic anhydrase inhibitors in conditions such as glaucoma, epilepsy, and cancer, these findings provide a foundation for developing next‐generation inhibitors with improved pharmacological profiles.

Furthermore, the observed antioxidant and antimicrobial activities suggest that isoindolinone derivatives could serve as promising candidates for multi‐target therapeutic strategies, addressing multiple pathological conditions simultaneously. Future research should focus on elucidating the underlying molecular mechanisms driving these interconnected bioactivities and further optimizing these compounds through structural modifications and in vivo studies. These efforts will be essential in determining their full therapeutic potential and facilitating their transition into clinical applications.

## 7 Experimental

### 7.1 General Remarks

All solvents, 2‐benzoylcarboxylic acid, and alcohols used in this study were commercially available and utilized without further purification. IR spectra were recorded using a Perkin‐Elmer spectrophotometer with 0.1 mm cells in CHCl_3_. ^1^H and ^13^C NMR spectra were obtained on a Bruker spectrometer operating at 400 MHz and 100 MHz, respectively, with chemical shifts reported in parts per million (ppm). Mass spectra were recorded using an Agilent QTOF (Quadrupoletime‐of‐flight) spectrometry device. Melting points were determined using a Gallenkamp melting‐point apparatus (WA11373).

### 7.2 General Procedure Synthesis of Novel Isoindolinone (2a‐f) Derivatives

Chlorosulfonyl isocyanate (1.1 eq) was added to a solution of 2‐benzoylbenzoic acid (1.0 eq) and a catalytic amount of trifluoroacetic acid in 10 mL of dichloromethane, and the mixture was stirred at room temperature for 2 h. Subsequently, 1 mL of the corresponding alcohol (ROH) was added, and stirring was continued at room temperature for 1 h. After the reaction, volatiles were removed under reduced pressure. The resulting residue was purified via thin‐layer chromatography (TLC) using a mixture of ethyl acetate and *n*‐hexane (1:4) as the eluent, yielding the pure product.

**Ethyl1‐hydroxy‐3‐oxo‐1‐phenylisoindoline‐2‐sulfonate** (**2a**): White solid (0,70 g, 95%), m.p 128°C–130°C; ^**1**^ **H‐NMR** (400 MHz, CDCl_3_): δ=7.86 (d, J: 7,7 Hz, 1H), 7.68 – 7.53 (m, 5H), 7.46–7.36 (m, 3H), 6.31 (bs, 1H), 4.23 (m, 2H), 1.35 (t, *J* = 7.1 Hz, 3H). ^**13**^ **C‐NMR** (100 MHz, CDCl_3_): *δ*= 168.4, 146.9, 138.1, 134.6, 131.0, 130.2, 129.5, 125.8, 125.7, 125.2, 124.7, 94.4, 68.8, 14.7. **IR (CHCl**_**3**_, **cm** ^**−1**^ **):** 3229, 2963, 1772, 1435, 1352, 1263, 1154, 1102. **HRMS (ESI):** calcd for C_16_H_16_NO_5_S [(M + H)^+^]: 334.3655; found: 334.3661.

**Propyl1‐hydroxy‐3‐oxo‐1‐phenylisoindoline‐2‐sulfonate** (**2b**): White solid (0,71 g, 92%), m.p 132°C–134°C; ^**1**^ **H‐NMR** (400 MHz, CDCl_3_): δ=7.86 (d, J: 7,7 Hz, 1H), 7.69–7.35 (m, 8H), 6.54 (bs, 1H), 4.18–3.94 (m, 2H), 1.78–1.67 (m, 2H), 0.92–0.85 (m, 3H). ^**13**^ **C‐NMR** (100 MHz, CDCl_3_): δ= 168.4, 134.6, 130.9, 130.1, 129.4, 129.3, 129.1, 125.8, 125.7, 125.3, 124.6, 94.5, 73.9, 22.4, 10.2. **IR (CHCl**_**3**_, **cm** ^**−1**^ **):** 3242, 2965, 1758, 1443, 1359, 1263, 1172, 1066. **HRMS (ESI):** calcd for C_17_H_18_NO_5_S [(M + H)^+^]: 348,0900; found: 348,0912.

**Isopropyl1‐hydroxy‐3‐oxo‐1‐phenylisoindoline‐2‐sulfonate** (**2c**): White solid (0,69 g, 90%), m.p 136°C–138°C; ^**1**^ **H‐NMR** (400 MHz, CDCl_3_): δ=7.98 (d, J: 7,3 Hz, 1H), 7.66‐7.31 (m, 8H), 6.58 (bs, 1H), 4.71–4.65 (m, 1H), 1.26 (m, *J* = 6.2 Hz, 6H). ^**13**^ **C‐NMR** (100 MHz, CDCl_3_): δ= 164.1, 143.8, 137.6, 135.3, 131.1, 130.1, 129.9, 126.6, 125.9, 125.4, 125.0, 85.0, 79.7, 22.9. **IR (CHCl**_**3**_, **cm** ^**−1**^ **):** 3226, 2963, 1757, 1448, 1385, 1263, 1178, 1085. **HRMS (ESI):** calcd for C_17_H_18_NO_5_S [(M + H)^+^]: 348,0900; found: 348,0905.

**Butyl1‐hydroxy‐3‐oxo‐1‐phenylisoindoline‐2‐sulfonate** (**2d**): White solid (0,74 g, 93%), m.p 141°C–143°C; ^**1**^ **H‐NMR** (400 MHz, CDCl_3_): δ=7.87–7.85 (m 1H), 7.68–7.53 (m, 5H), 7.43–7.37 (m, 3H), 6.25 (bs, 1H), 4.24–4.10 (m, 2H), 1.71–1.64 (m, 2H), 1.42–1.33 (m, 2H), 0.94–0.90 (m, 3H). ^**13**^ **C‐NMR** (100 MHz, CDCl_3_): δ= 168.2, 147.0, 138.1, 134.6, 130.9, 130.2, 129.5, 125.7, 125.2, 124.6, 94.4, 72.4, 30.8, 18.8, 13.8. **IR (CHCl**_**3**_, **cm** ^**−1**^ **):** 3247, 2968, 1756, 1469, 1364, 1262, 1192, 1125. **HRMS (ESI):** calcd for C_18_H_20_NO_5_S [(M + H)^+^]: 362,1057; found: 362,1068.

**Isobutyl1‐hydroxy‐3‐oxo‐1‐phenylisoindoline‐2‐sulfonate** (**2e**): White solid (0,72 g, 90%), m.p 139°C–141°C; ^**1**^ **H‐NMR** (400 MHz, CDCl_3_): δ=7.88 (d, J: 7,3 Hz, 1H), 7.69–7.39 (m, 8H), 6.09 (bs, 1H), 4.00–3.85 (m, 2H), 2.06–1.95 (m, 1H), 0.98 (d, J = 7.1 Hz, 6H). ^**13**^ **C‐NMR** (100 MHz, CDCl_3_): δ= 168.2, 147.1, 138.3, 134.6, 130.9, 130.1, 129.5, 125.8, 125.2, 124.5, 94,3, 74.8, 28.2,18.9. **IR (CHCl**_**3**_, **cm** ^**−1**^ **):** 3245, 2968, 1772, 1467, 1392, 1265, 1156, 1125. **HRMS (ESI):** calcd for C_18_H_20_NO_5_S [(M + H)^+^]: 362,1057; found: 362,1063.

**Cyclohexyl1‐hydroxy‐3‐oxo‐1‐phenylisoindoline‐2‐sulfonate** (**2 f**): White solid (0,73 g, 86%), m.p 114°C–116°C; ^**1**^ **H‐NMR** (400 MHz, CDCl_3_): δ=8.08 (d, J: 7,3 Hz, 1H), 7.70–7.31 (m, 8H), 4.15–4.03 (m, 1H), 1.77– 1.74 (m, 2H), 1.59–1.57 (m, 2H), 1.40–1.07 (m, 6H). ^**13**^ **C‐NMR** (100 MHz, CDCl_3_): δ= 166.9, 135.9, 133.4, 132.3, 131.5, 131.1, 130.6, 130.1, 129.6, 129.3, 128.8, 94.4, 78.2, 32.6, 25.4, 23.9. **IR (CHCl**_**3**_, **cm** ^**−1**^ **):** 3225, 2943, 1736, 1426, 1324, 1236, 1161, 1057. **HRMS (ESI):** calcd for C_20_H_21_NO_5_S [(M + H)^+^]: 387,1140; found: 387,1136.

### 7.3 CAI and CAII Inhibition Assay

Human erythrocyte carbonic anhydrase isoenzymes I and II were purified with a simple one‐step method using Sepharose‐4B‐l‐tyrosine‐sulfanilamide affinity chromatography [31]. This method is based on previously described protocols. The protein concentration of the eluates was determined spectrophotometrically at 595 nm using the Bradford method with bovine serum albumin as the standard protein [32]. The purity of the enzyme fractions was assessed by SDS‐PAGE on 8% slab gels, following the procedure described by Laemmli [33]. The esterase activity of the purified human erythrocyte carbonic anhydrase isoforms I and II were measured according to the method outlined by Verpoorte et al. [34]. The assay involved monitoring the increase in absorbance of the reaction medium using a spectrophotometer at 348 nm [35]. Acetazolamide was used as a reference drug for comparison in the inhibition assays. The inhibitory effects of test compounds on CA isoenzymes were evaluated by measuring the change in enzyme activity in the presence of the inhibitors compared to controls.

### 7.4 Antioxidant Assays

#### 7.4.1 ABTS^.+^ Scavenging Assay

Antioxidant capacity was determined using the ABTS radical cation scavenging method ABTS and potassium persulfate (K_2_S_2_O_8_) were mixed in equal proportions and incubated at room temperature to generate the radical cation [36]. The resulting solution was diluted with ethanol, and the absorbance of the control was adjusted to 0.928 ± 0.025 at 734 nm. Different concentrations of the compounds were prepared, added to test tubes, and adjusted with 0.2 mL of ethanol. To each tube, 0.8 mL of the ABTS radical solution was added. After incubation for 30 min, the absorbance of the remaining ABTS^•+^ was measured at 734 nm.

#### 7.4.2 Fe^3+^ Reducing Power Assay According to the FRAP Method

The ability of the carbamate compounds to reduce was evaluated using the FRAP assay [37]. Various concentrations of the compounds were dissolved in deionized water to a final volume of 0.2 mL in test tubes. To each tube, 500 µL of potassium ferrocyanide [K_3_Fe(CN)_6_] and 500 µL of phosphate buffer (0.2 M, pH 6.6) were added. The mixture was vortexed and incubated for 20 min at 50°C. Then, 500 µL of 10% trichloroacetic acid was added, and 500 µL of the upper layer of the solution was mixed with 100 µL of 0.1% FeCl_3_ and 500 µL of distilled water. The absorbance of the reducing effect was recorded using a spectrophotometer at 700 nm.

### 7.5 Antimicrobial Research

In the biological activity tests, two fungal strains (*Candida albicans ATCC 10231, Yarrowia lipolytica ATCC 20460*) two Gram‐positive (Gr + ) bacterial strains (*Bacillus subtilis ATCC 6633, Staphylococcus aureus ATCC 25923*), and two Gram‐negative (Gr−) bacterial strains (*Escherichia coli ATCC 43895, Klebsiella pneumoniae ATCC 13883*), were obtained from the culture collection of the Ataturk University Microbiology Laboratory. The bacterial strains were cultured on Luria‐Bertani agar (LBA) at 37°C, while the yeast strains were cultured on potato dextrose agar (PDA) at 30°C. After 24 h of incubation, the isolates were used in the subsequent studies.

### 7.6 Disc Diffusion Analysis

The bacterial inoculum was prepared according to the 0.5 McFarland standard, and a smear culture was performed on Petri dishes for the disk diffusion test, one of the most commonly used antibacterial assays. The antimicrobial susceptibilities of bacterial and fungal isolates were assessed by placing synthesized compounds at a concentration of 1000 μg/mL (1 μg active ingredient + 900 μL dH₂O + 100 μL DMSO) on Petri dishes, impregnating them onto disks, and measuring the zone diameters formed after 24 h of incubation at 37°C. All tests were conducted in triplicate. Nystatin and Ampicillin were used as a positive control, while DMSO was used as a negative control [38].

### 7.7 Cytotoxicity and Anticancer Activity

L929 (fibroblast cell line, ATCC CCL‐1) was cultured in DMEM/F‐12 medium with containing of 10% fetal bovine serum (FBS), 100 U/ml penicillin/streptomycin, A549 (lung cancer cell line, ATCC CCL‐185) were cultured in Dulbecco's modified eagle medium‐high glucose (DMEM‐high glucose) supplemented with 10% fetal bovine serum (FBS), 100 U/ml penicillin/streptomycin at 37°C in 5% CO_2_ incubation. The culture medium was changed every 2 days.

In vitro cytotoxic effects and anticancer activity of test compounds were performed by WST‐1 assay with following the standard protocol [39]. A concentration of 2×10^4^ cells/mL was cultured into 96‐well plates and incubated at 37°C at 5% CO_2_ for 24 h. Cells were exposed to test substances at concentrations of 6.25 μg/ml‐ 500 μg/ml in serum‐free medium for 24 h. Cell viability was measured at 450 nm on a multiplate reader (Thermo Scientific) after incubation at 37°C for 2 h with the addition of WST‐1 (120 μL/well). Morphological examinations of cells were observed under the inverted microscope. Cell viability was calculated according to the formula as below;

(%)Viability=([(Absorbanceoftreatedcell)−(absorbanceofblank)])/([(Absorbanceofcontrol)−(absorbanceofblank))×100

## Author Contributions

Yusuf Serdar Yazıcıoğlu: Formal analysis. Şeydanur Elmas: Formal analysis, validation. Zeynep Kılıç: Formal analysis. Murat Çelik: Data curation. Buket Bakan: writing—review and editing, writing—original draft, validation, investigation. Ufuk Atmaca: Investigation, funding acquisition, writing—original draft, writing—review and editing, visualization, validation, Formal analysis, supervision, resources. Songül Bayrak: supervision, writing—review and editing, writing—original draft, validation, formal analysis.

## Supporting information


**Supporting Information Summary**.

## Data Availability

The data that support the findings of this study are available from the corresponding author upon reasonable request.
