# Supplementary material for: Synthesis of Novel Isoindolinones: Carbonic Anhydrase Inhibition Profiles, Antioxidant Potential, Antimicrobial Effect, Cytotoxicity and Anticancer Activity
Source: J Biochem Mol Toxicol. 2025 Apr 14;39(4):e70261. doi: 10.1002/jbt.70261 (PMC11995831; doi:10.1002/jbt.70261)
Supplement: Supplementary file 1 — Supporting Information Summary. [file JBT-39-e70261-s001.docx]

**Supporting Information Summary**

Experimental procedures, characterization data of products, ^1^H and ^13^C NMR spectra
